# Manager–Team (Dis)agreement on Stress-Preventive Behaviours: Relationship with Psychosocial Work Environment and Employees’ Well-Being

**DOI:** 10.3390/ijerph21080989

**Published:** 2024-07-28

**Authors:** Stefano Toderi, Glauco Cioffi, Joanna Yarker, Rachel Lewis, Jonathan Houdmont, Cristian Balducci

**Affiliations:** 1 Department of Psychology, University of Bologna, 40127 Bologna, Italy; stefano.toderi@unibo.it; 2 Affinity Health at Work, London SW12 9NW, UK; j.yarker@bbk.ac.uk (J.Y.); rachel.lewis@bbk.ac.uk (R.L.); 3 Birkbeck Business School, University of London, London WC1E 7HU, UK; 4 Faculty of Medicine & Health Sciences, University of Nottingham, Nottingham NG7 2RD, UK; jonathan.houdmont@nottingham.ac.uk; 5 Department for Life Quality Studies, University of Bologna, 47921 Rimini, Italy; cristian.balducci3@unibo.it

**Keywords:** self–other agreement, management competencies, work-related stress, polynomial regression, psychosocial work environment, well-being

## Abstract

The “Management Competencies to Prevent and Reduce Stress at Work” (MCPARS) approach focuses on identifying the stress-preventive managers’ competencies able to optimise the employees’ well-being through the management of the psychosocial work environment. Considering leadership as contextualised in complex social dynamics, the self–other agreement (SOA) investigation of the MCPARS may enhance previous findings, as it allows for exploring the manager–team perceptions’ (dis)agreement and its potential implications. However, no studies have tested the MCPARS using the SOA and multisource data. Grounded in Yammarino and Atwater’s SOA reference theory, we conducted an in-depth investigation on the MCPARS’s theoretical framework by examining the implications of manager–team (dis)agreement, regarding managers’ competencies, on employees’ psychosocial environment (H1–H2) and affective well-being (H3). Data from 36 managers and 475 employees were analysed by performing several polynomial regressions, response surface, and mediation analyses. The results reveal a significant relationship between SOA on MCPARS and employees’ perceptions of the psychosocial environment (H1). Employees report better perceptions when supervised by *in-agreement good* or *under-estimator* managers, while lower ratings occur under *over-estimator* or *in-agreement poor* managers (H2). Moreover, the psychosocial environment significantly mediated the relationship between SOA on MCPARS and employees’ well-being (H3). The MCPARS theoretical model’s soundness is supported, and its implications are discussed.

## 1. Introduction

Ensuring health and promoting well-being while creating quality employment opportunities are two critical objectives set out by the ONU Agenda 2030 to achieve global sustainable development (Goals 3 and 8). In line with these issues, a recent report by the World Health Organization [1], recognising that work significantly impacts our lives, emphasised how psychosocial risks and work-related stress pose two of the most challenging health concerns.

Psychosocial risks are considered the primary causes of stress [2]. They were defined in 1986 by the International Labour Organization [3] in terms of the interactions among job content, work organisation, and management, on the one hand, and the employees’ competencies and needs on the other. Risks emerge when critical work factors (i.e., psychosocial factors, such as job demands, autonomy, work-life balance, and role clarity, among others) are inadequately managed, leading to negative consequences often observed across three levels: individual (e.g., sleep disorders, anxiety, cardiovascular disorders); organisational (e.g., poor productivity, absenteeism, turnover); social (e.g., Gross Domestic Product, healthcare service costs) [4]. Conversely, an optimised management of these factors can create a work environment that enhances well-being and performance [5]. It is well-known that the creation of a psychologically healthy workplace is closely connected to line managers’ actions, and the approach developed by Yarker et al. [6,7,8] called *Management Competencies for Preventing and Reducing Stress at Work* (MCPARS) explicitly considers the employees’ well-being promotion through the management of the psychosocial factors as a leadership task. This manuscript, by applying the concept of the self–other agreement (SOA), aims to investigate the MCPARS’s theoretical framework with a multisource and multi-prospective design. To do this, we first present the MCPARS- and SOA-relevant literature; then, we derive specific hypotheses based on the past research and explain how we address them empirically.

### 1.1. Management Competencies to Prevent and Reduce Stress at Work (MCPARS)

The MCPARS is a competence-based approach developed to be integrated into leadership development interventions and wider people practices. It was designed to address psychosocial risks by increasing the managers’ awareness of their well-being-oriented behaviours through self-assessment exercises and upward feedback exposure (team rating). The authors considered the indirect effect of supervisors on employees’ well-being through psychosocial factors [9,10] and focused on supervisors’ behaviours devoted to managing the psychosocial work environment. This latter, indeed, is an extension of the *Management Standard* approach [10], which outlines ‘states to be achieved’ as ideal work-related situations in six key stressor areas (i.e., demands, control, support, relationships, role, and organisational change), referring to critical psychosocial factors. Following extensive qualitative and quantitative research, the MCPARS identifies four Key Management Competencies [i.e., *Respectful and Responsible (RR)*, *Managing and Communicating existing and future Work (MCW)*, *Reasoning and managing Difficult Situations (RDS)*, and *Managing the Individual within the Team (MIT)*] essential to manage the psychosocial work environment effectively and to optimise employee well-being. Some evidence supports the soundness of MCPARS’s theoretical framework. Toderi et al. [11], with a single-source and cross-sectional design, found the four management competencies linked to the six psychosocial factors identified by the *Management Standards* approach [10]. A multilevel study demonstrated that supervisors’ self-assessed stress-preventive management competencies were linked to employees’ affective well-being through the mediating influence on the employees’ perceived psychosocial work environment [12]. Additionally, Houdmont et al. [13] found that when employees reported working with a manager with low stress-preventive management competencies, this was associated with elevated odds of psychological distress, low resilience, and low work engagement. While Chenevert et al. [14], investigating the relationship between workplace bullying and post-traumatic stress disorder symptomology, highlighted that an employee supervised by a manager with high stress-preventive management competencies reported less workplace bullying perceptions and a weakened indirect effect of role conflict and exposure to bullying on PTSD symptomology. Another study in the field highlighted that the supervisor’s *Respectful and Responsible* competence was found to be crucial to increase work engagement and employees’ performance [15], suggesting the importance of further research on the relationship between MCPARS’s theoretical framework and performance-oriented outcomes.

However, the potential implications of manager–team (dis)agreement regarding stress-preventive behaviours were never explored in relation to the expected outcomes (i.e., psychosocial environment and well-being). This information is crucial for assessing the MCPARS approach, highlighting how the agreement–disagreement level with proposed behaviours may affect employees.

### 1.2. Measurement of Management Behaviour and the Role of the Self–Other Agreement

Self-ratings of leadership skills alone are not good predictors of a leader’s effectiveness [16,17]. New leadership models and theories no longer describe leadership as an individual characteristic. Instead, they tend to present conceptualisations of the phenomenon based on dyadic, shared, and relational aspects contextualised in complex social dynamics [18,19]. Researchers generally agree on leadership as jointly established by leaders and followers [20,21,22]. Thus, obtaining multisource data involving multiple social actors’ perceptions is recommended to better investigate leader effectiveness and outcomes [23]. There has been much focus on whether leaders’ perceptions of their leadership behaviours overlap with, or diverge from, the perceptions of their subordinates, peers, or superiors (e.g., [16,17,24,25]). This theme was cemented when Yammarino and Atwater published a seminal paper on the self–other agreement in 1997 [25]. The authors suggested that leaders who either agree or disagree with their followers on their leadership style can be descriptively categorised as *over-estimator*, *under-estimator*, *in-agreement good leader*, *and in-agreement poor leader.* Different traits, development needs, and organisational outcomes characterise each. Specifically, the *over-estimators* encompass those managers who appraise their competencies more favourably when compared to the appraisal provided by their employees, whereas *under-estimators*, conversely, undervalue their skills compared to how their collaborators perceive them. In contrast, managers who align their self-ratings with those of their team are defined as *agreement managers* and divided into *good* when the shared rating is high and *poor* when it is low.

Much of the current interest in SOA research derives from two primary factors: (a) It is posited to be an indicator of self-awareness, and (b) it is related to several outcomes of interest, including leader effectiveness and derailment (for a review, see [16]). Scholars who support the link between the SOA and self-awareness (in leadership research) claim that the discrepancies between self-ratings and others’ ratings allow for a rare insight into a leader’s interpersonal world [26] and that self-awareness is having an accurate understanding of one’s strengths and weaknesses; thus, it can be measured by comparing self and others’ ratings of leaders’ behaviours [27,28]. Wohlers and London [29] appear to be the first in the literature to operationalise self-awareness by examining how participants’ self-ratings compared with others’ ratings. It is now common to assume that if a leader’s self-ratings are congruent with others’ ratings, the leader is more self-aware than those whose ratings are incongruent (e.g., [16,29,30,31,32,33,34,35,36,37,38,39,40,41]). Moreover, recent research has bolstered the viewpoint that SOA category membership yields significant implications for outcomes related to both organisational and well-being aspects, as well as for training paradigms for leaders that utilise feedback exposure [16,17,39,42,43,44,45], supporting the second SOA factor of interest (i.e., the link between SOA and leadership effectiveness).

### 1.3. The Present Study

To the best of our knowledge, the SOA has not been applied to the examination of “healthy leadership” models. This study is timely, as models of “healthy leadership” are garnering increasing interest in occupational health science [46] because the positive outcomes of traditional performance-oriented approaches may not fully capture the specific behaviours relevant to the health and well-being of employees [47,48]. By applying the concept of the self–other agreement, this paper aims to investigate the potential implications of managers’ awareness of their behaviours by testing the *Management Competencies to Prevent and Reduce Stress at Work*’s theoretical framework [6,7]. Although the MCPARS approach and intervention protocol have been acknowledged by EU-OSHA and Eurofound [49,50] as an excellent practice for manager development, the need for further research has been recognised [47]. Thus, a multisource and multi-prospective investigation could contribute to advancing this approach. To do this, we followed the Flenoor et al. [16] recommendations to use widely accepted methods when SOA is being treated as a predictor by performing several polynomial regressions with response surface analysis, as suggested in the SOA literature [51,52,53].

#### Hypotheses Development

Given that Transformational Leadership appears to be the most dominant concept in leadership research, it is unsurprising that it has been the most extensively studied leadership model in the SOA literature [17]. In a sample of 38 mental health teams, Aarons et al. [44] investigated the relationship between SOA on Transformational Leadership (TL) and organisational culture and found that culture suffered more when supervisors rated themselves more positively than employees. Teams supervised by *over-estimator* managers reported significantly worse organisational culture perceptions regarding all three sub-dimensions accounted in the study (i.e., subservience, consensus, and conformity) than teams supervised by *under-estimator* managers. Tekleab et al. [40] reported that SOA on TL was related to employees’ perceptions of job satisfaction and leader effectiveness. Recently, Hasson et al. [45] highlighted that SOA on TL was related to employees’ learning climate perceptions, and that teams supervised by *in-agreement good* managers reported better perceptions of the learning climate than teams supervised by *in-agreement poor* managers. Zhao et al. [54], by investigating the relationship between manager–team (dis)agreement on TL and team performance, revealed that team performance was higher when both the manager and the teams’ perceptions of the leader’s TL capabilities were high (i.e., *in-agreement good* manager) rather than low (i.e., *in-agreement poor* managers). However, they did not find a significant difference regarding team performance (rated by teams) between employees working with *under-estimator managers* and those working with *over-estimator* managers. Moreover, a study that computed a score for (dis)agreement found that disagreement on TL was related to high conflict and burnout, and low social support, engagement, and health [55].

Considering other leadership styles, Amundsen and Martinsen [42] investigated the link between SOA on empowering leadership and job satisfaction, turnover intention, and leader effectiveness in 50 Norwegian municipal teams. The findings revealed that employees working with *under-estimator* managers reported better job satisfaction than the teams working with *over-estimator* managers. Additionally, the turnover intention of employees supervised by *over-estimator* mangers was significantly higher than that of employees working with *under-estimator* managers. Concerning job satisfaction and turnover intention, no significant results were found regarding the difference between teams supervised by *in-agreement good* and those supervised by *in-agreement poor* managers. However, considering SOA on empowering leadership and leader effectiveness, in-*agreement poor* managers were rated as the most ineffective leaders in this sample. The SOA on empowering leadership was also found to be related with the self-leadership of employees [40].

Another leadership style investigated with the SOA methodology is the contingent reward [45]. Regarding this style, teams working with *in-agreement good* managers reported a better learning climate than teams working with *in-agreement poor* managers. Additionally, the same study highlighted that employees’ empowerment perceptions were higher for teams working with *under-estimator* managers than teams supervised by *over-estimator* managers on their contingent reward capabilities.

The SOA on Leader–Member Exchange (LMX) leadership was investigated by Ertutk et al. [56]. This study highlighted that *under-estimator* managers received the best rating from their subordinates on their LMX capabilities, followed by *in-agreement good managers*, and, lastly, *in-agreement poor* and *over-estimator managers*. Moreover, these ratings were related to subordinates’ perceptions regarding their supervisors’ performance.

Overall, the SOA research suggests that the level of (dis)agreement between the supervisor’s and subordinates’ ratings on supervisor behaviours is linked not only to leader effectiveness [25] but also to the organisational climate and, more specifically, to employees’ perception and outcomes (for reviews [16,17]). However, no study has explored the SOA on leadership skills explicitly dedicated to well-being and its potential implications for employees’ well-being outcomes. A recent systematic review by Rudolph et al. [46] highlighted that scholars have developed various health-oriented leadership models over the past decade, and further contributions on this topic will follow.

Thus, considering the well-established relationship between leader self-awareness (measured employing the SOA) with several positive outcomes, such as follower trust and organisational commitment [57], mentoring behaviour [58], performance (e.g., [25,54,59,60]), leader effectiveness, and follower satisfaction [40], we expect a significative relationship between the self–other (dis)agreement on the four stress-preventive management competencies and the psychosocial work environment perceptions of employees. The first hypothesis formulated is:

**H1:**  Self–other agreement between managers’ and employees’ assessment of the managers’ stress-preventive management competencies will be associated with subordinates’ perceptions of the psychosocial work environment.

Additionally, taking into account the positive association of both *in-agreement good* and *under-estimator managers* with numerous organisational and individual outcomes, and the highly to moderately adverse outcomes for *over-estimators* and *in agreement poor managers* [16,17,25,40,42,44,54,59], we suggest a specific hypothesis on the relationship between SOA on stress-preventive management competences and the psychosocial work environment reported by employees. Namely, we expect that:

**H2:**  Employees will report the highest rating of the psychosocial environment if coordinated by *in*-*agreement good* and *under-estimator* managers and the lowest rating if the employees are led by *in-agreement poor* or *over-estimator* managers.

To further investigate the potential implications of manager–team (dis)agreement on managers behaviours, considering (1) the well-established relationship between psychosocial work environment and stress-related outcomes [12,61,62,63], and (2) the MCPARS’s theoretical framework (i.e., MCPARS’s competencies->psychosocial work environment->well-being) [6,7], we further investigate the mediating role of the psychosocial work environment between SOA on stress-preventive management competences and employees’ well-being:

**H3:**  The psychosocial work environment will mediate the relationship between SOA on stress-preventive management competencies and the job-related well-being outcomes.

**Figure 1:**
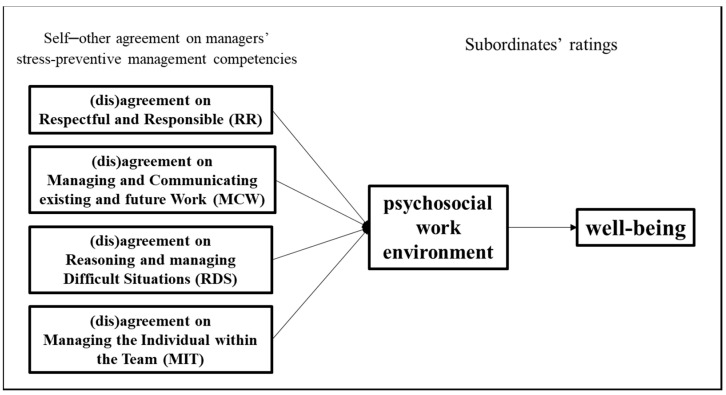
Proposed research conceptual model. Please note that mediating variable (psychosocial work environment) and outcome variable (well-being) are measured as subordinates’ perceptions.

The conceptual model tested in the present study is reported in Figure 1.

In doing so, we contribute first to the MCPARS literature, by proposing a deep investigation of the competencies outlined by the model, adopting multisource and multi-perspective data and investigating for (dis)agreement implications, following the leadership research best practices recommendation suggested by Avolio et al. [23] and the SOA literature [16,17,25], respectively. Secondly, within the context of the SOA literature, we propose an initial exploration of the healthy leadership model and linked organisational outcomes (e.g., the psychosocial work environment and well-being).

## 2. Materials and Methods

### 2.1. Participants and Procedure

The initial sample comprised 549 employees (subordinates) and 40 managers (supervisors) of Italian Public Administration. Data were collected for an organisational well-being survey in 2018, then implemented with an organisational supervisor-focused intervention for workplace health. Some participants were excluded because they were the only employee associated with a supervisor, or due to incomplete measures. This resulted in a final analytic sample of 475 subordinates (female = 56.4%; male = 16.2%; no answer = 27.4%) and 36 supervisors, which comprised 36 teams with a mean of 13.19 members in each team (range 4–54; SD = 12.63).

### 2.2. Measures

*Stress-preventive management competencies* were measured by the short 36-item version of the Stress Management Competencies Indicator Tool (SMCIT; [11,64]), measuring each of four closely related competencies with nine items. The short version of this tool has repeatedly demonstrated psychometric soundness (e.g., [13]) and allows brevity in the survey design where it is needed rather than the full 66-item version of the SMCIT [7]. The first competence, *Respectful and Responsible (RR)*, included items such as “Acts calmly in pressured situations”. The second competence, focusing on *Management and Communication existing and future Work (MCW)*, featured items like “Deals with problems as soon as they arise”. The third competence, *Reasoning and Managing Difficult Situations (RDS)*, involved items like “Acts as a mediator in conflict situations”. The fourth competence, *Managing the Individual within the Team (MIT)*, included items such as “Tries to see things from my point of view”. Similarly to the original 66-item version, the questionnaire has two different forms: one intended for the self-assessment of supervisors (all items are prefixed by “I… ”) and one for employees (“My supervisor…”). The supervisor/employee is requested to indicate her or his agreement with each of the presented statements on a five-point Likert scale (1—strongly disagree; 5—strongly agree). Cronbach’s alpha for SMCIT and other measures used are reported in Table 1.

*Psychosocial work environment* was investigated by using the short 25-item version of the Stress Management Indicator Tool (SIT; [65,66,67]), which measures the following six psychosocial factors: demands, control, support, relationships, role, and change. Items are rated on a 5-point Likert scale, varying from 1 (strongly disagree, according to specific items) to 5 (strongly agree). Only employees were asked to fill out this measure. Example items are “I have unachievable deadlines” (demands, four items) and “I have some say over the way I work” (control, four items). Where necessary, items were reverse-scored prior to analysis so that higher scores on each psychosocial factor indicated a better psychosocial work environment. As the four competencies play a direct role in managing psychosocial factors [6,7,11] and considering that the SIT questionnaire allows for an overall measurement of the environment [65], in the analysis, a single overall measure was adopted, reflecting the quality of the psychosocial work environment, with higher scores indicating a better environment.

*Well-being* was measured using the Italian Adaptation of Warr’s job-related affective well-being scale (JAWB; [68,69]). The scale consists of 12 feelings, six positive (contented, calm, relaxed, enthusiastic, cheerful, optimistic) and six negatives (depressed, tense, uneasy, gloomy, worried, miserable). Respondents (i.e., employees) were asked to evaluate each feeling, indicating how often, over the last weeks, their job had made them feel in that way (1—never to 5—always). An overall scale score was derived for the analyses by reversing the negative item, thus measuring the overall positive affective job experiences.

### 2.3. Statistical Procedure

Our research hypotheses propose that the (dis)agreement between two variables (supervisor-rated and subordinate-rated leadership behaviours, i.e., self–other agreement) predicts immediate (i.e., psychosocial work environment) and distal (i.e., well-being) outcomes. (Dis)agreement has been examined using absolute or squared difference scores computed as the quantity of (in)congruence of two predictor variables. Nevertheless, those analytical methods have been profoundly critiqued for various reasons (e.g., [52]). Investigating the (in)congruence without considering the measures separately as supervisor-rated and subordinate-rated variables, it is difficult to address whether the supervisor’s perceptions determine the outcome, the subordinate’s perception, or their (in)congruence. Incongruence in one direction (i.e., X > Y) may have different effects than incongruence in the other direction (X < Y). Hence, we followed the recommendations for (in)congruence studies by performing a polynomial regression with Response Surface Analysis [51,52,53,70] to test our hypothesis. This analysis enables us to examine the combined impact of two variables on a third while at the same time retaining information about the differences between the variables. This data analytic approach aggregates employees’ ratings to the team level (K = 36) to make team-level inferences about relationships among variables (see also [43,44,45]). To justify aggregation of the provider data to the team level, intraclass correlation coefficients (ICC(1)s) and within group agreement (rWG(j)) statistics were calculated; these are presented in Table 1. As it can be seen in Table 1, the (ICC(1)s) were all positive and significant, and the mean rWG(j)s were above 0.80 for all scales. Overall, the analyses support the aggregation of subordinates’ ratings.

Furthermore, we followed the three-step procedure outlined by Shanock et al. [70] to examine SOA, also recommended by Gibson, Cooper, and Conger [71]. The first step consists of exploring agreement and disagreement between supervisors and collaborators to confirm whether the level of disagreement was sufficient to warrant further analysis. Fleenor et Prince al. [72] suggested at least 10% of disagreement to make further analyses meaningful. The disagreement was defined as at least 0.5 SD of the standardised mean score on the two predictors, and in order to classify the leaders into the four categories, we followed the Nielsen et al. [43] (2022) procedure. Thus, we standardised the score for self and followers, and a supervisor with a standardised score on the self-rating half a standard deviation above their subordinates’ scores was categorised as an *over-estimator*. A supervisor with a standardised self-rated score half a standard deviation below their subordinates’ scores was categorised as an *under-estimator*. Supervisors within these limits were categorised as in agreement with subordinates [73]. Supervisors who were in agreement and rated by their team above the mean score were classified as *in-agreement good*, whereas supervisors who agreed with their subordinates but rated by the team below the sample mean were classified as *in-agreement poor.* This classification, based on theory, is only used for descriptive purposes and to aid interpretation of the response surface analysis and is not used in the polynomial regressions where continuous variables are used. In the second step, four polynomial regression analyses were conducted, one for each of the four stress-preventive management competencies. These analyses were performed on scale-centred variables to aid interpretation of the findings [51]. The psychosocial work environment was regressed on supervisors’ rating (X), subordinates’ rating (Y), the cross product of supervisors’ and subordinates’ rating (XY), and the square of supervisors’ (X^2^) and subordinates’ ratings (Y^2^) of management behaviours. Suppose the predictors explain significant variance in the outcome variable (i.e., R^2^ of the polynomial regression is significant). In that case, further analyses are justified, which include calculating the four surface test values, α1, α2, α3, and α4, based on unstandardised regression coefficients [74].

The surface test values were plotted in 3D graphs in the third step. The four surface test values represent the slopes and curvature of two lines. The first line (X = Y), the “line of perfect agreement”, runs diagonally from the nearest to the farthest corners of the graph. α1 is the slope along the “line of perfect agreement” and represents how the agreement between the predictors relates to the outcome. α2 is the curvature along the line for perfect agreement and shows whether this relationship (between agreement and outcome) is linear or non-linear. The second line (X = −Y), called the “line of incongruence”, runs diagonally from the left to the right corner. The slope along the line of incongruence is reflected by α3 and the curvature by α4. When the slope along the congruence or incongruence line is significant and positive, we can conclude that (in)congruence at high levels of management behaviours results in higher outcomes than at low levels (i.e., *in-agreement good* and *over-estimator* managers). Concurrently, when the slope is significant and negative, we can conclude that higher outcomes are for low levels (i.e., *in-agreement poor* and *under-estimator* managers).

Overall, to test **H2**, we used the response surface analysis, while to test **H1** and **H3**, we used the block variable approach suggested by Edwards and Cable [75]. We combined the five polynomial terms (X, Y, X^2^, X × Y, and Y^2^) into a block variable, a weighted linear composite, in which the weights are the estimated regression coefficient in the polynomial regression. The block variable does not change the total explained variance [76,77]. Once we built the block variable, we rerun the regressions, calculated the standardised regression coefficient to test **H1**, and performed a mediation analysis from SOA to well-being (mediating by psychosocial environment) to test **H3**. We estimated bias-corrected confidence intervals for the indirect effects by bootstrapping 20,000 samples. Preliminary descriptive, correlational analyses were conducted using SPSS 22 (IBM, Armonk, NY, USA) and its PROCESS macro for the mediation [78]. Response surface analyses were performed following Shanock et al. [70] guidelines.

## 3. Results

The descriptive statistics and bivariate correlations are reported in Table 2. The correlations between managers and employees’ ratings of the four management competencies behaviours were all non-significant, indicating that variation exists between the ratings of self and others and that perceptual (in)congruence analyses were warranted. Concurrently, both self-reported scores on stress-preventive management competencies and scores from direct reports’ evaluations positively correlated in all four stress-preventive management competencies. As expected, the psychosocial work environment perceptions and well-being demonstrated a positive relationship.

In line with the procedure outlined by Shanock et al. [70], we first analysed the level of agreement between supervisors’ and subordinates’ perceptions of leadership. The discrepancies in leader and follower ratings were greater than 10% [72], warranting polynomial regression analyses (see Table 3).

Moreover, the four polynomial regressions, one for each competency, were all significant, as was their respective block variable regression on the psychosocial work environment (see Table 4 and Table 5). Thus, self–other agreement on stress-preventive management competencies explained a significant variance in psychosocial work environment perceived by teams. The standardized path coefficient (*ß*) for the block variable of (dis)agreement on RR predicting the psychosocial work environment perceptions was 0.80 (*p* < 0.001); for MCW, it was 0.85 (*p* < 0.001); for RDS, it was 0.90 (*p* < 0.001); and for MIT, it was 0.77 (*p* < 0.001); fully supporting **H1**. We therefore calculated the surface test values, α1-α4, which are presented in Table 4.

**Figure 2:**
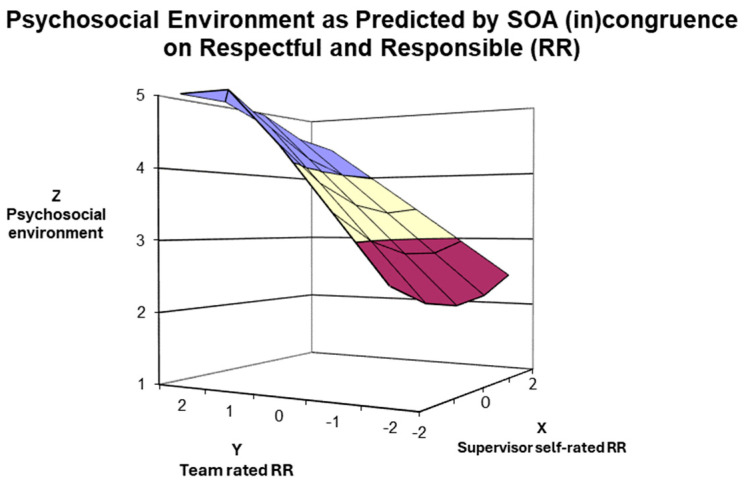
Surface Response Analysis for the influence of SOA on Respectful and Responsible (RR) Competence on the Psychosocial Work Environment.

Hypothesis 2 proposed that the direct employees of *in-agreement*, *good* and *under-estimator* managers would perceive a better psychosocial environment perception than subordinates of *in-agreement poor* and *over-estimator* managers. The result of the polynomial surface test highlighted that the slope along the line of perfect agreement for *Respectful and Responsible* (RR) competence was positive and significant (α1 = 0.50; t = 2.643; *p* = 0.009), suggesting that subordinates of *in-agreement good* managers reported a better psychosocial environment than those of *in-agreement poor* managers. Further, the slope along the line of incongruence was negative and significant (α3 = −0.89; t = −6.294; *p* < 0.001), indicating that subordinates of under-estimator managers perceived a better psychosocial environment than those of over-estimator managers. As shown in Figure 2, the highest psychosocial environment perceptions of subordinates were found for *in-agreement good* and *under-estimator* managers. In contrast, lower ratings were found for *over-estimator* and *in-agreement poor* managers.

**Figure 3:**
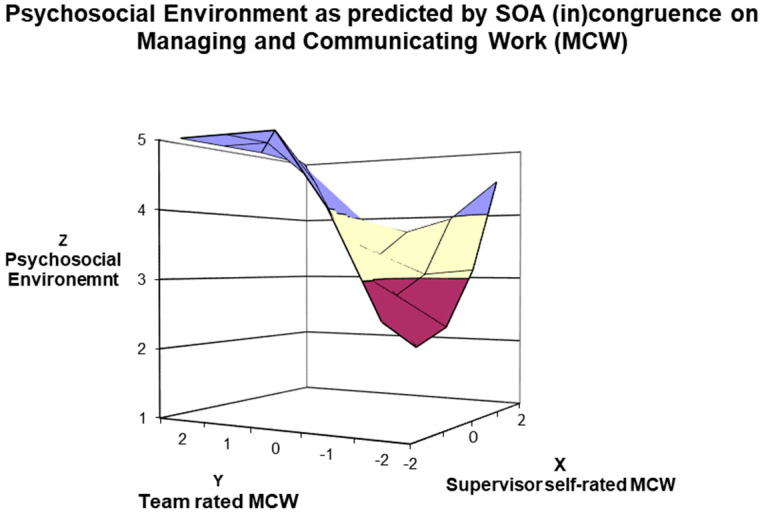
Surface Response Analysis for the influence of SOA on Managing and Communicating Work (MCW) Competence on the Psychosocial Work Environment.

A similar pattern was found for self–other agreement on *Managing and Communicating existing and future Work* (MCW) competence. Results demonstrated that agreement between managers’ and employees’ ratings of MCW significantly influenced psychosocial environment perceptions (α1 = 0.50, t = 2.699, *p* = 0.008). Specifically, the greatest level of psychosocial environment was found when the supervisor and subordinates similarly rated MCW highly (i.e., *in-agreement good* managers). Moreover, the analysis revealed that the direction of discrepancy between managers’ and employees’ ratings of MCW significantly influenced psychosocial environment perceptions (α3 = −1.52, t = −8.125, *p* < 0.001). Consistent with our second hypothesis, the psychosocial environment management was greater when employees’ ratings of MCW were high and managers’ self-ratings of MCW were low (i.e., under-estimators) than when employees’ ratings of MCW were low and manager self-ratings of MCW were high (i.e., *over-estimators*). However, incongruence between supervisors’ and subordinates’ ratings of MCW had a significant curvilinear relationship with consensus (α4 = 1.00, t = 8.248, *p* < 0.001), such that as ratings between managers and employees became increasingly discrepant, ratings of the psychosocial environment increased. As it can be seen in Figure 3, psychosocial environment perceptions were highest for the subordinates of in-agreement good and under-estimator managers, followed by *over-estimator* managers, and they were lowest for *in-agreement poor* managers concerning MCW competence.

**Figure 4:**
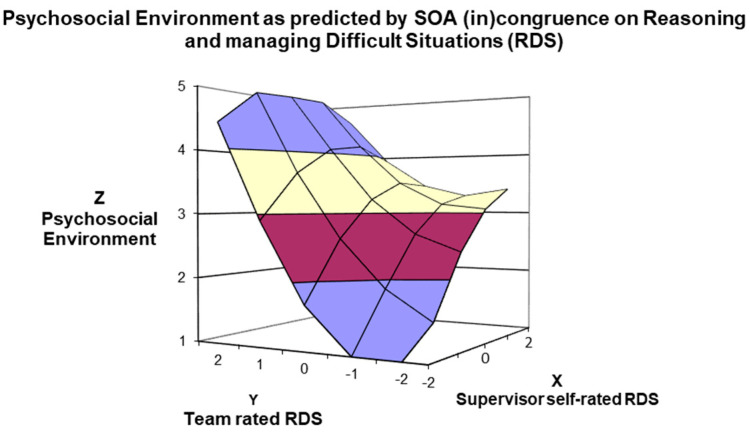
Surface Response analysis for the influence of SOA on Reasoning and Managing Difficult Situation (RDS) Competence on the Psychosocial Work Environment.

The third competence considered was *Reasoning and managing Difficult Situations* (RDS). The slope was positive and significant along the line of perfect agreement (α1 = 1.15; t = 14.74; *p* < 0.001) and negative and significant along the line of disagreement (α3 = −0.25; t = −5.617; *p* < 0.001), suggesting better management skills for *in-agreement good* than for *in-agreement poor* mangers, and for *under-estimator* than for *over-estimator* managers. Moreover, in the line of congruence, the curve was significantly negative (α2 = −0.21; t = −37.61; *p* < 0.001), suggesting a concave/downward relation. Specifically, when ratings of RDS were congruent and low, ratings of the psychosocial environment were low; the line of congruence increased sharply as the agreed ratings became moderate to high and then decreased a little as the ratings became extremely high. Concurrently, as for MCW, the incongruence between managers’ and employees’ ratings of RDS had a significant curvilinear relationship with the psychosocial work environment (α4 = 0.17; t = 2.822; *p* = 0.005), such that as the ratings between managers and teams became increasingly discrepant, psychosocial work environment perceptions increased. Figure 4 displays the best psychosocial work environment perceptions for employees of *in-agreement good* managers, then *under-estimators*, followed by *over-estimators* and, lastly, by *in-agreement poor* managers regarding the RDS competency.

**Figure 5:**
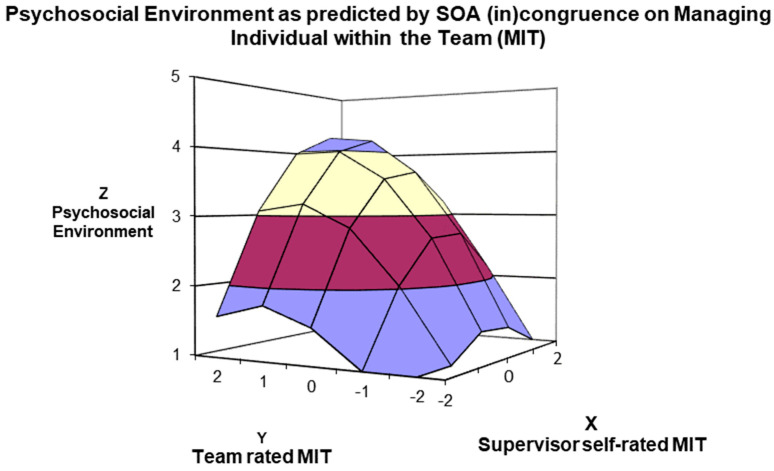
Surface Response analysis for the influence of SOA on Managing the Individual within the Team (MIT) Competence on the Psychosocial Work Environment.

The results of the last surface analysis investigating the relationship between *Managing the Individual within the Team* (MIT) self–other agreement on the psychosocial work environment suggested better ratings for *in-agreement good* than for *in-agreement poor* managers (α1 = 1.08; t = 12.048; *p* < 0.001), and for *under-estimators* than for *over-estimators* (α3 = −0.23; t = −2.575; *p* = 0.01), as revealed by the (in)congruence coefficient slopes’ significance and sign. Moreover, the curve along the line of agreement was significant and negative (α2 = −0.43; t = −11.913; *p* < 0.001), suggesting a concave relationship between agreement and the psychosocial environment, as for RDS. To conclude, the surface along the incongruence line significantly curved downward (α4 = −0.61; t = −6.287; *p* < 0.001). All surfaces are plotted in 3D surface graphs (see Figure 2, Figure 3, Figure 4 and Figure 5). Overall, the findings supported H2. As presented in Figure 5, the highest psychosocial environment perceptions were reported by the teams of *in-agreement good* managers, then for *under-estimator* managers, followed by *in-agreement poor* and *over-estimator* managers on MIT competence.

**Figure 6:**
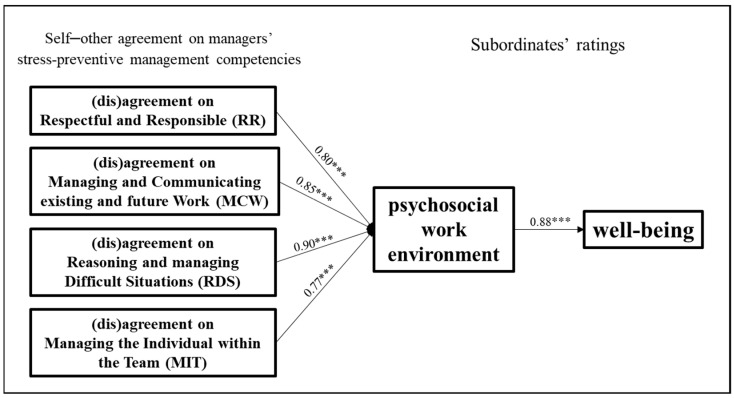
Direct effects of the proposed research conceptual model. Note: *** = *p* < 0.01.

Finally, to test **H3**, the mediating effect of the psychosocial work environment on the relationship between self–other agreement on the MCPARS’s competencies and the well-being of employees, we used the block variable approach ([75]; see also [56]). The path coefficient from the psychosocial work environment to well-being is significant (*ß* = 0.88, *p* < 0.001). Moreover, as shown in Table 5, the indirect effects from (dis)agreement on Management Competencies on well-being via the psychosocial work environment were all significant since no confidence intervals included zero. Thus, **H3** was validated by the findings. See Figure 6 for a graphical representation of the proposed conceptual model’s direct effect results.

## 4. Discussion

With this research, we aimed first to contribute to MCPARS’s approach and extend the current research on how the four stress-preventive management competencies, outlined by the MCPARS’s theoretical framework [6,7], might affect employees’ psychosocial work environment and well-being perceptions by examining the self–other agreement (SOA). Secondly, we wanted to contribute to the SOA literature by proposing an initial exploration of the healthy leadership model (never explored before) and related organisational outcomes (i.e., psychosocial work environment and well-being, in our case). Lastly, by following SOA research recommendations (e.g., [16,51,52,53,75]), we further examined the suggested methodological approach by employing polynomial regression coupled with response surface analysis instead of alternative methodologies such as difference scores or agreement categorisation.

Overall, three key findings emerged: two regarding the MCPARS’s theoretical framework and one regarding the detailed implications of the level of (dis)agreement on the psychosocial environment perceptions reported by the employees.

First, the results highlighted a significant relationship between the SOA on the four MCPARS and teams’ psychosocial work environment perceptions. These findings suggest a general (dis)agreement effect of MCPARS’s competencies (multisource rated) on the psychosocial environment perceptions by the employees. Previous findings highlighted how this direct link was supported [11,12]. Following the best practices recommendations for leadership research by using multisource data [16,23] and the SOA literature, our results contribute to exploring the MCPARS approach’s soundness even when considering leadership in a complex multi-perspective social dynamic [18,19]. Moreover, this outcome is in line with previous research, which outlined a positive link between manager–team agreement on leadership style (e.g., transformational, empowering) with several positive outcomes, such as trust, performance, leader effectiveness, and the job satisfaction of employees [40,54,57,59,60].

Second, the SOA on the four competencies considered in our study significantly indirectly affected the job-related affective well-being of employees. This analysis still brings new knowledge about the robustness of MCPARS’s theoretical model in its intentions to identify the stress-preventive competencies that can indirectly impact the well-being of employees through the mediated role of the psychosocial work environment. These findings are coherent with a study that employed a multilevel design and managers’ self-assessment of the MCPRAS’s competencies [12].

Third, the response surface analysis results depicted the role of SOA membership implications for supervised employees for both agreement and disagreement lines. The congruence (i.e., agreement) investigation highlighted that higher ratings on the psychosocial work environment perceptions were significantly linked to higher rating congruence (i.e., *in-agreement good* managers). The incongruence (i.e., disagreement) investigation findings suggested a higher psychosocial work environment perception (employees’ rating) for managers who underestimate their management competencies than for managers who overestimate their well-being-devoted behaviours. These results can be seen in Figure 2, Figure 3, Figure 4 and Figure 5, in which the 3D surface graphs evidence that the direct subordinates of *in-agreement good* and *under-estimator* managers provided the highest perception scores (regarding psychosocial work environment) for all four stress-preventive competencies. Based on these findings, we can infer that, in line with other studies, *in-agreement good* (e.g., [40,45,54]) and *under-estimator* (e.g., [42,44,56]) managers confirm their excellent relationship with the desired outcomes of the leadership model under consideration. However, our results reveal additional information indicating that this relationship is not solely linked to the performance-oriented models typically used in self–other agreement research but also to well-being-oriented managers’ behaviours.

Moreover, the 3D graphs in Figure 3 and Figure 4 provide further insights into how manager–team (in)congruence regarding the four MCPARS competencies affects perceptions of the psychosocial work environment and might bring to psychosocial risks for employees. The teams supervised by *in-agreement poor* and *over-estimator* mangers on the MCPARS’s competencies reported the lowest ratings of the psychosocial work environment and can be equally hazardous for employees. However, regarding the competence of *Managing and Communicating existing and future Work* (see Figure 3) and the competence *in Reasoning and managing Difficult Situations* (see Figure 4), the teams reporting lower perceptions of the psychosocial environment are those supervised by *in-agreement poor* managers, and only afterwards by *over-estimator* managers. This finding slightly contrasts with research on performance-oriented models, which rarely reported that teams supervised by *over-estimator* managers reported the worst outcomes of interest (some exceptions [45,54]). Thus, we can infer that in our sample, being supervised by an *over-estimator* manager in the competencies of *Managing and Communicating existing and future Work* and *Reasoning and managing Difficult Situations* is not as risky as being supervised by *in-agreement poor* managers. A possible interpretation of these results is that *in-agreement poor* managers, even if aware of their capabilities, are unwilling or unable to change their behaviour due to low self-esteem and/or self-efficacy [40]. Overall, our results suggest some practical implications.

### 4.1. Practical Implications

Supervisors deeply affect the well-being of employees both directly, through the emotional contagious mechanism [79,80], and indirectly, through the management of psychosocial factors [9,10]. Considering their pivotal role in the intervention process, organisational development, and change [5,47], developing good managers is a must for organisations to create a sustainable work environment and is highly recommended [81,82]. In line with this best practice, our study offers important implications for team managers and organisational health and human resource practitioners.

First, managers should develop strategies to foster high-level agreement on the four stress-preventive management competencies outlined by the MCPARS’s theoretical framework. Our findings support the idea that being aware of not being a good manager (i.e., *in-agreement poor* manager) is insufficient to prevent psychosocial risks for employees, and the same is true for *over-estimator* managers. The achievement of manager–team high-level agreement on MCPARS’s competencies should improve the employees’ psychosocial work environment perceptions and, indirectly, the team’s well-being. Some strategies could be team-building activities, which may help foster shared views, or team internal behaviourally oriented discussion concerning the management of the psychosocial factors (e.g., demands, role clarity, autonomy, etc.) to increase managers’ awareness of their management style and understand the changes needed. However, independently seeking feedback is not an easy task for managers. Thus, organisational health and human resource practitioners must develop or refine skills to make superiors more aware of their management styles by creating a scaffolding organisational feedback culture. This aim might be achieved by intervention focused on teams and/or managers. The intervention on teams was never explored before. Concerning managers training activities, the MCPARS’s approach already suggested two intervention protocols [8]. The first involves the engagement of employees in data collection and, therefore, the possibility of providing upward feedback to managers. The second, so-called “self-reflection” modality, excludes the participation of employees and focuses exclusively on managers’ perceptions. Regarding the first modality, some critical concerns should be highlighted. Negative upward feedback (i.e., high managers’ self-assessment compared to subordinates’ low ratings; *over-estimator* managers) can reduce a leader’s commitment to their subordinates [83] and might bring anger and discouragement reactions [84]. A possible strategy to tackle this issue could be to design personalized coaching activities [85]. This may help *over-estimator* managers to develop a more realistic self-image and *in-agreement poor* managers to develop self-efficacy. The second modality could reduce the disagreement between manager and team by enhancing reflective personal leadership [86], in which leaders reflect on their own leadership practices through self-assessment exercises on MCPARS’s competencies or group discussions to share possible obstacles, peers’ suggestions, and best practices.

### 4.2. Limitations

Although the present results support the use of self–other agreements (SOA) in the study of employees’ well-being, it is appropriate to recognise several potential limitations. The first limitation concerns the adoption of SOA as an indicator of managers’ self-awareness in aiming to investigate the impact of their behaviour on employees. Scholars agree on the SOA operationalisation to measure manager’s self-awareness regarding their capabilities exists [16,27,28,29,30,31,32,33,34,35,36,37,38,39,40,41]. However, classical theorists of self-awareness denote that self-awareness consists of two key components: first, an understanding of oneself and, second, the ability to anticipate how others perceive one [39,87,88]. Research on leadership has consistently explored the first component, employing the self–other agreement (SOA) to highlight the manager’s awareness in terms of understanding their strengths and weaknesses (i.e., self-understanding). Interestingly, the second component of self-awareness was rarely investigated (i.e., prediction—other). For instance, a study by Taylor et al. [39] showed that the prediction of others’ ratings on managerial competencies, performed by managers taking their subordinates’ points of view, explained a greater percentage of variance in leader effectiveness than self–other ratings. This further line of enquiry was beyond the scope of this study. Furthermore, a second limit, shared with similar studies (e.g., [12,39,44]), is the cross-sectional nature of our data. This method limits the degree to which we can make causal inferences regarding the relationships between SOA and well-being.

## 5. Conclusions

The main contributions of the present study are threefold. First, by employing polynomial regression, response surface, and mediation analyses, we examined the combined effect (as well as the rating difference effect) of both perspectives on the manager’s stress-preventive competencies (self and other; manager and team) on the related outcomes (psychosocial environment and well-being). Overall, we can conclude that our findings support the MCPARS’ theoretical framework and enhance the reliability of the approach under consideration. Second, the results revealed remarkable differences among the four SOA mangers’ categories (i.e., *over*; *under*; *good*; *poor*) [25]. Teams supervised by *in-agreement good* and *under-estimator* managers reported the best perceptions of the psychosocial work environment, followed by *over-estimator* and, lastly, *by in-agreement poor* managers. With these results we can conclude that following the methodological approach suggested by the SOA research paper milestone [51,52,70,75] is necessary for (in)congruence investigation.

Finally, being mindful of the limit of employing the SOA in self-awareness operationalisation, we support the view that the critical issue is not whether the agreement is an index of self-awareness by itself but, rather, whether disagreement affects changes in self-awareness and suggests the need for behaviour change [27]. Considering that organisational interventions aimed at increasing superiors’ self-awareness of the management style adopted are strongly supported [35], we suggest that, in line with Nielsen et al. [43], future directions should take into account the SOA membership [25] in leadership training (which includes exposure to employee feedback) to improve intervention outcomes and research findings.

## Figures and Tables

**Table 1:** Intraclass Correlation Coefficient (ICC), Within Group Agreement rWG(j), and Cronbach’s alpha of study measures.

Measure	n° Items	ICC	Mean rWG (j)	Cronbach’s α
Respectful and Responsible	9	0.38 *	0.88	0.86
Managing and Communicating Work	9	0.45 *	0.88	0.89
Reasoning and Managing Difficult Situations	9	0.60 *	0.90	0.93
Managing the Individual within the Team	9	0.38 *	0.92	0.90
Psychosocial Environment	25	0.29 *	0.98	0.93
Well-being	12	0.40 *	0.93	0.91

Note: * *p* ≤ 0.001.

**Table 2:** Means, standard deviations (SD), and correlations among study variables.

	Mean (SD)	1	2	3	4	5	6	7	8	9
**1. Respectful and Responsible (Manager)**	1.11 (0.33)	-								
**2. Respectful and Responsible (Team)**	0.82 (42)	0.17	-							
**3. Managing and Communicating Work (Manager)**	1.00 (0.30)	0.58 ***	−0.06	-						
**4. Managing and Communicating Work (Team)**	0.63 (0.48)	0.05	0.79 ***	0.09	-					
**5. Reasoning and managing Difficult Situations (Manager)**	0.89 (0.48)	0.62 ***	−0.07	0.75 ***	−0.08	-				
**6. Reasoning and managing Difficult Situations (Team)**	0.48 (0.54)	0.02	0.78 ***	0.06	0.92 ***	−0.07	-			
**7. Managing the Individual within the Team (Manager)**	0.87 (0.46)	0.54 **	0.09	0.67 ***	0.14	0.56 **	0.11	-		
**8. Managing the Individual within the Team (Team)**	0.64 (0.49)	0.11	0.77 ***	0.03	0.84 ***	−0.09	0.80 ***	0.30	-	
**9. Psychosocial Environment (Team)**	3.82 (0.33)	0.14	0.81 ***	0.11	0.82 ***	−0.02	0.85 ***	0.21	0.78 ***	-
**10. Well-being (Team)**	3.36 (0.39)	0.09	0.43 **	−0.04	0.36 *	−0.11	0.40 *	−0.01	0.27	0.59 ***

Note: *** *p* ≤ 0.001; ** *p* ≤ 0.01; * *p* ≤ 0.05.

**Table 3:** Percentage of self–other agreement membership in our sample.

Self-Other Agreement Category Membership	*In-Agreement Good*	*In-Agreement Poor*	*Over-Estimator*	*Under-Estimator*
**Respectful and Responsible (RR)**	22.2%	22.2%	33.3%	22.2%
**Managing and Communicating Work (MCW)**	36.1%	16.7%	19.4%	27.8%
**Reasoning Difficult Situations (RDS)**	13.9%	16.7%	30.6%	36.1%
**Managing the Individual within the Team (MIT)**	19.4%	13.9%	33.3%	33.3%

**Table 4:** Polynomial regression analysis and surface values results.

Psychosocial Environment Predicted by Self–Other Agreement				
Competence	Respectful and Responsible (RR)	Managing and Communicating Work (MCW)	Reasoning and Managing Difficult Situations (RDS)	Managing the Individual within the Team (MIT)
	**B**	**B**	**B**	**B**
**Constant**	3.50 ***	3.58 ***	3.22 ***	3.55 ***
**X (b_1_)**	−0.19	−0.51 **	0.45 ***	0.42 ***
**Y (b_2_)**	0.70 ***	1.01 ***	0.70 ***	0.65 ***
**X^2^ (b_3_)**	0.09	0.30 ***	−0.16 ***	−0.30 ***
**XY(b_4_)**	−0.09	−0.51 ***	−0.19 ***	0.09
**Y^2^ (b_5_)**	−0.01	0.19 ***	0.14 ***	−0.22 ***
**F**	164.16 ***	241.32 ***	387.23 ***	137.39 ***
**R^2^**	0.64	0.73	0.81	0.59
**Surface test**				
**α1 = (b_1_ + b_2_)**	0.50 **	0.50 **	1.15 ***	1.08 ***
**α2 = (b_3_ + b_4_ + b_5_)**	−0.01	−0.02	−0.21 ***	−0.43 ***
**α3 = (b_1_ − b_2_)**	−0.89 ***	−1.52 ***	−0.25 ***	−0.23 **
**α4 = (b_3_ − b_4_ + b_5_)**	0.18	1.00 ***	0.17 **	−0.61 ***

Notes: X = Supervisor self-rating; Y = Team rating; α1 = slope of agreement line; α2 = curve of agreement line; α3 = slope of disagreement line; α4 = curve of disagreement line; *** *p* ≤ 0.001; ** *p* ≤ 0.01; * *p* ≤ 0.05.

**Table 5:** Results from tests effects of (dis)agreement on stress-preventive management competencies on the psychosocial work environment (direct effect) and well-being (indirect effect, via psychosocial work environment).

Block Variable (Dis)agreement	Direct Effect on Psychosocial Work Environment	Indirect Effect on Well-Being via Psychosocial Work Environment [95% Bootstrapped Confidence Intervals]
**Respectful and Responsible (RR)**	0.80 ***	0.69 [BootLLCI = 0.61; BootULCI = 0.76]
**Managing and Communicating Work (MCW)**	0.85 ***	0.74 [BootLLCI = 0.63; BootULCI = 0.83]
**Reasoning and managing Difficult** **S** **ituations (RDS)**	0.90 ***	0.79 [BootLLCI = 0.65; BootULCI = 0.93]
**Managing the Individual within the Team (MIT)**	0.77 ***	0.68 [BootLLCI = 0.60; BootULCI = 0.77]

Notes: Standardised coefficients are reported: BootLLCI, lower level of 95% confidence interval; BootULCI, upper level of 95% confidence interval. *** *p* ≤ 0.001.

## Data Availability

The data were collected under a convention between the University of Bologna and the organisations, which permits the publication of research results but not the complete dataset. Consequently, the data are not publicly accessible due to privacy concerns.
